# End-to-End Implementation of Various Hybrid Neural Networks on a Cross-Paradigm Neuromorphic Chip

**DOI:** 10.3389/fnins.2021.615279

**Published:** 2021-02-02

**Authors:** Guanrui Wang, Songchen Ma, Yujie Wu, Jing Pei, Rong Zhao, Luping Shi

**Affiliations:** Department of Precision Instrument, Center for Brain-Inspired Computing Research (CBICR), Beijing Innovation Center for Future Chip, Optical Memory National Engineering Research Center, Tsinghua University, Beijing, China

**Keywords:** hybrid neural networks, cross-paradigm computing, neuromorphic chip, mapping framework, end-to-end implementation

## Abstract

Integration of computer-science oriented artificial neural networks (ANNs) and neuroscience oriented spiking neural networks (SNNs) has emerged as a highly promising direction to achieve further breakthroughs in artificial intelligence through complementary advantages. This integration needs to support individual modeling of ANNs and SNNs as well as their hybrid modeling, which not only simultaneously calculates single-paradigm networks but also converts their different information representations. It remains challenging to realize effective calculation and signal conversion on the existing dedicated hardware platforms. To solve this problem, we propose an end-to-end mapping framework for implementing various hybrid neural networks on many-core neuromorphic architectures based on the cross-paradigm Tianjic chip. We construct hardware configuration schemes for four typical signal conversions and establish a global timing adjustment mechanism among different heterogeneous modules. Experimental results show that our framework can implement these hybrid models with low execution latency and low power consumption with nearly no accuracy degradation. This work provides a new approach of developing hybrid neural network models for brain-inspired computing chips and further tapping the potential of these models.

## Introduction

Neural networks have been widely used to deal with intelligence problems. In general, they can be divided into non-spiking artificial neural networks (ANNs) (Lecun et al., 2015) and spiking neural networks (SNNs) (Maass, 1997; Ghosh-Dastidar and Adeli, 2009). These two types of neural models are distinct in information representation and processing. In ANNs, information is propagated with multi-valued data. Intensive representation makes ANNs achieve high accuracies in a myriad of tasks, such as image classification (He et al., 2016), speech recognition (Lam et al., 2019), and action recognition (Wu et al., 2016). In contrast, SNNs encode information in event-driven binary spike trains. Through internal neuron dynamics to memorize spatio-temporal information, SNNs show advantages in various scenarios with rich temporal information and sparse data streams (Shi et al., 2017; Haessig et al., 2018; Wu et al., 2019). Owing to their different advantages, in recent years there is a growing trend of integrating ANNs and SNNs to explore hybrid neural networks (HNNs) toward artificial general intelligence (Marblestone et al., 2016; Zhang et al., 2016; Ullman, 2019). For example, in some cases of event-driven tasks (Srinivasan and Roy, 2019; Lee et al., 2020), researchers use SNN modules for abstracting sparse temporal information, and further combine ANN modules for improving the classification performance. Similarly, in some cases of static image processing tasks (Kheradpisheh et al., 2018; Chancán et al., 2020), researchers use ANN modules to extract the edge contrasts in images and further process them with SNN modules for low power consumption. Besides, ANNs and SNNs also work collaboratively to perform complex tasks in Pei et al. (2019); Yang et al. (2019).

Hybrid neural networks have a promising perspective on the development of artificial general intelligence. However, by far these models are mainly studied and implemented on general-purpose platforms (i.e., CPU or GPU) (Kheradpisheh et al., 2018; Srinivasan and Roy, 2019; Chancán et al., 2020; Lee et al., 2020). On the other side, HNNs retain the basic properties of neural networks, being promising in high-efficiency implementation on domain-specific hardware platforms (Sze et al., 2017). However, their unique cross-paradigm mechanisms, such as the mixed dataflow of multi-valued data and spike trains, hinder the implementation on dedicated platforms, thereby slowing down the exploration of diverse cross-paradigm integration. Thus, it is highly expected to develop a general scheme of implementing HNNs on dedicated platforms for high efficiency, which can facilitate the iteration of software and hardware co-optimization and eventually promote the development of hybrid neural models.

There are two challenges in implementing HNNs on dedicated hardware platforms. The first is to support the simultaneous execution of ANN and SNN computing paradigms. In the traditional ANN or SNN field, each has its respective hardware platforms to support their efficient execution, e.g., deep learning accelerators for ANNs (Chen et al., 2014; Han et al., 2016; Jouppi et al., 2017; Chen et al., 2019) and neuromorphic chips for SNNs (Furber et al., 2014; Merolla et al., 2014; Davies et al., 2018). However, due to the significant differences between ANNs and SNNs in terms of information representation, computation philosophy and memory organization, the basic operators and data transmission methods of these two types of platforms are incompatible. Therefore, neither of the above hardware platforms can simultaneously support the execution of ANNs and SNNs, which impedes the implementation of HNNs. The second is the hybrid data interactions between ANNs and SNNs. In HNNs, the hybrid data interaction modules connect ANNs and SNNs, which have a non-negligible impact on the performance of the models when implemented on a hardware platform. Usually, the hybrid data interaction results in at least two-fold computational costs: (1) realizing signal conversion between multi-valued data and spike trains will bring extra resource consumption and execution delay; (2) the signal conversion and timing configuration will in turn affect the resource consumption and execution time of ANN and SNN modules. In current dedicated hardware platforms, signal conversion at the input interface needs to be implemented when the external data cannot match the information format transmitted and processed internally. Therefore, extra devices and resources are usually required, such as “spike generator” (Esser et al., 2016; Shukla et al., 2019) or “frame maker” (Shukla et al., 2019). However, this separative method will not only destroy the continuity of hardware execution to a certain extent, but also make it difficult to comprehensively measure and evaluate the implementation cost of signal conversion and network computing via a unified standard.

In this paper, we provide a systematic scheme of implementing HNNs on many-core neuromorphic architectures based on software-hardware cooperation from the perspectives of hardware features and mapping framework. First, we use a new type of cross-paradigm Tianjic chip (Pei et al., 2019) as the hardware infrastructure. From the aspects of basic operations, communication method, and timing execution mechanism, the hardware features that support the implementation of HNNs are abstracted. On this basis, we propose an end-to-end mapping framework to implement HNNs on many-core neuromorphic chips. Inspired by the modular approach, we divide the implementation of HNNs into the pure computing modules of single ANNs and SNNs, and the signal conversion modules between them. The pure computing modules can be realized by using the existing single-paradigm mapping methods (Esser et al., 2013; Deng et al., 2018; Ji et al., 2018). To realize the hybrid data interactions between ANNs and SNNs, configuration schemes for four typical signal conversions methods are established. Besides, we also develop a global timing adjustment mechanism to match the different working periods among these modules. Taking some HNN models as examples, we analyze their performance in terms of resource overhead, running speed, and energy consumption when deployed on the Tianjic chip. This implementation framework provides a generic approach for developing hybrid neural models through the hardware-software collaboration.

The rest of this paper is organized as follows. Section “Hardware Infrastructure” introduces the basic operation, communication format, and timing schedule mechanism of the Tianjic chip from the perspective of hardware feature abstraction. Section “End-to-End Mapping Framework” shows the characteristics of neural networks’ execution on many-core neuromorphic chips, and presents the proposed end-to-end mapping framework for hybrid neural models. The resource overhead, timing analysis, and energy consumption of the example hybrid networks are reported in Section “Experimental Results.” Finally, we come up with the overall conclusions and carry out further discussions in Section “Conclusion and Discussion.”

## Hardware Infrastructure

**FIGURE 1:**
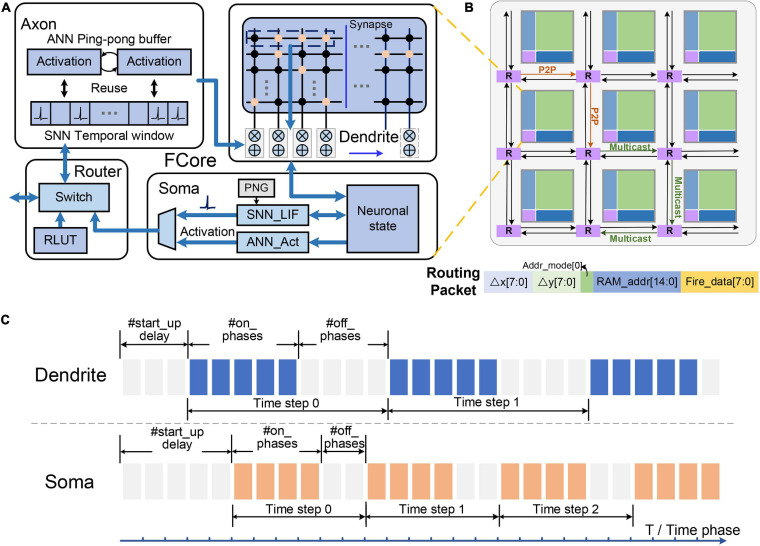
Illustration of the Tianjic chip architecture: **(A)** fine-grained configurable operation modules; **(B)** unified communication format; **(C)** adjustable timing schedule.

Tianjic adopts a unified, configurable, and scalable architecture to support cross-paradigm computing, which provides a general platform for the separative execution or hybrid computing of ANNs and SNNs. In this section, we will briefly introduce the overall architecture of the Tianjic chip (see Figure 1), including its basic operation, communication format, and timing schedule mechanism which support our mapping framework.

### Fine-Grained Configurable Operation Modules

Functional core (FCore) is the basic unit of the Tianjic chip, which consists of four modules, including an axon for input organization, a dendrite (with synapses) for integration operations, a soma for non-linear neuronal transformation, and a router for activation transmission (Figure 1A). Each module can be configured to work in different modes or perform different operations, which enables the chip to support both ANN and SNN models. Among these modules, the dendrite and the soma are the main computing engines. Equipped with the synapse memory, the dendrite constitutes a 256 × 256 virtual crossbar, which can realize various vector and matrix operations. Table 1 lists the vector and matrix operations used in this paper, including vector-matrix multiplication (*VMM*), vector-vector accumulation (*VVA*) and vector buffering (*VB*).

**TABLE 1:** Integration and transformation operations in Tianjic.

	Mode	Definition
Dendrite operation	*VMM*	*y* = *W⋅x*
	*VVA*	$y=\sum_{i}x_{1}+x_{2}+\ldots +x_{n}$
	*VB*	*y* = *x*
Soma transformation	*LUT_fun*	*y* = *f*(*u* + b)
	*LIF_fun*	Leaky-integration-and-fire operation
Membrane potential	*change*	Update membrane potential after soma transformation
	*keep*	Membrane potential keeps unchanged after soma transformation

The calculation results of the dendrite are updated into a memory shared with the soma and the soma generates the neuronal output according to the updated membrane potential. By combining some basic calculations, the soma can realize a variety of non-linear transformations in different modes of ANNs and SNNs. In an ANN-mode soma, arbitrary activation function can be supported by a configurable lookup table (LUT). In an SNN-mode soma, its internal operation corresponds to the leaky-integration-and-fire (LIF) operation of spiking neurons. Furthermore, some more complicated operations, such as threshold adaption and random firing, can also be enabled through specific configurations. After the transformation of the soma, the update mode of the shared memory can be divided into *keep* or *change*, corresponding to whether the membrane potential stored therein will be changed according to the calculation result of the soma.

### Unified Communication Format

These FCores are connected by a multifunctional and scalable routing network, and arranged in a 2D mesh topology. The routing network is composed of the router and the axon in each FCore, which are responsible for sending and receiving information respectively. In the router, both artificial and spiking neurons transmit their information through a unified communication format. In addition to the address and control information in the traditional AER (Address-Event Representation) protocol (Mahowald, 1993), the routing packet can also carry different multi-valued data in this unified communication format. Specifically, this multi-valued data represents the efferent activation value of artificial neurons in the ANN mode and the state information of spiking neurons (e.g., membrane potential) in SNN mode. Notably, the router generates a packet only when a spike is generated and needs to be transmitted, which is in an even-driven manner.

As shown in Figure 1B, these data packets can be delivered to single or multiple arbitrary target FCores through point-to-point (P2P) or multicast routing. When arriving at the destination FCore, the packet is decoded to a binary spike or multi-valued activation according to the working mode of the local axon. In the ANN mode, the axon directly obtains multi-value activations from routing packets and store them in a ping-pong buffer. In the SNN mode, the axon stores spike trains of each input within a historical temporal window. Via a timing factor calculator (TFC), the spike trains can be weighted and summed according to the timing factors. Based on this cross-paradigm and unified data communication format, we can easily realize the basic connection structure that supports mixed dataflows as described in section “Execution of Neural Networks on Neuromorphic Chips.”

### Adjustable Timing Schedule

The timing execution mechanism in the Tianjic chip has two typical characteristics: the reconfigurable phase pattern in an execution time step and the independent working schedule of each FCore’s modules.

**Reconfigurable phase pattern in an execution time step:** There are two levels of execution period in Tianjic, which are time phase and time step. The time phase is a basic computational period to perform a round of computation, and the time step includes multiple time phases and therefore is a higher level of execution period. As shown in Figure 1C, the number of time phases in a time step and their on-off (enable and disable) pattern are controlled by timing registers (i.e., start-up delay, #on_phases and #off_phases). This configurable phase pattern provides a flexible support for matching different execution periods of ANNs and SNNs.

**Independent working schedule of each FCore’s modules:** In a time phase, the dendrite integrates the inputs stored in the axon and updates the membrane potential into the shared memory, meanwhile the soma performs non-linear transformation given the integrated membrane potential. In each FCore, the phase patterns of the dendrite and the soma can be configured independently. In this way, different timings of input and output processing can be implemented in the same FCore to perform signal conversion between spike trains and multi-valued activations.

## End-To-End Mapping Framework

Before introducing the mapping scheme, we briefly recall the execution mechanism of single-paradigm neural network models on many-core neuromorphic chips. Then, we introduce the main design features of our end-to-end mapping framework, which enable a high-performance mapping of HNNs on many-core neuromorphic chips. With configurable FCores, three basic connections are designed to support the mixed dataflows. By using the divide-and-conquer strategy, we further divide the implementation of HNNs into the pure computing modules of single ANNs and SNNs, and the hybrid data interaction modules between them. The pure computation can be implemented using the existing mapping methods for the single paradigm. To solve the problem of the hybrid data interaction, we construct the configuration schemes for typical signal conversion methods and a global timing adjustment mechanism between these different modules.

### Execution of Neural Networks on Neuromorphic Chips

**FIGURE 2:**
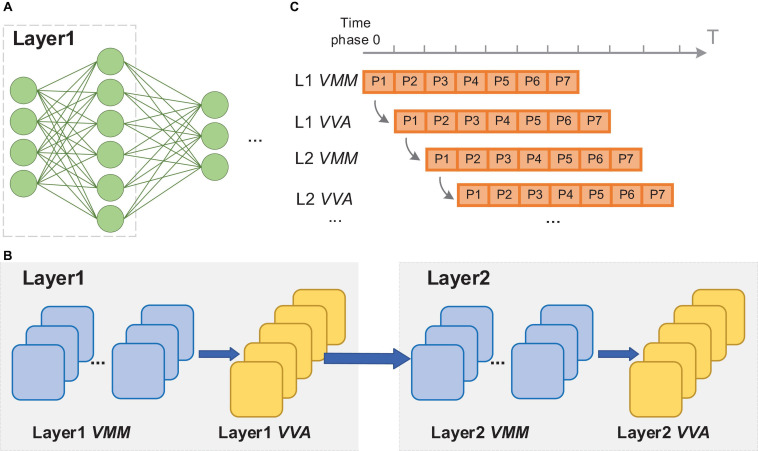
Illustration of the execution of the neural network on neuromorphic chips: **(A)** the example network structure; **(B)** the FCore groups after network mapping; **(C)** timing schedule of the FCore groups.

Generally, the implementation of neural networks on many-core neuromorphic chips is achieved by utilizing spatial mapping methods, in which the calculations in different layers are realized via the allocated FCore groups. These FCore groups continuously process the input data in a pipelined manner. Taking a fully connected network (Figure 2A) for illustration, we present this process in Figure 2. As shown in Figure 2B, in each layer, the calculations between input activations and weights are split into multiple spatial *VMM* operations due to the limited fan-in capability (the number of inputs a neuron can handle) and fan-out capability (the number of outputs a neuron can drive) of each FCore. Therefore, each *VMM* FCore obtains partial calculation results, and extra *VVA* FCores are required to accumulate the corresponding neurons’ partial states. In this layer-wise splitting manner, the workload of the original network will be mapped to a combination of FCore groups that perform different operations. Figure 2C exhibits the execution timing of these FCore groups, wherein each FCore performs the same operation repeatedly and continuously at every time phase. In addition, the data is continuously propagated and processed among FCore groups along the depth dimension of the network. When the calculation results are sent to the next FCore group for processing, the current FCore group can start the processing the following input sample at the same time, achieving an efficient pipelined processing. This inter-group pipeline brings high throughput, and is decoupled with the network depth. Most single-paradigms of ANNs and SNNs follow this method when implemented on many-core neuromorphic chips (Akopyan et al., 2015; Ji et al., 2018; Shao et al., 2019; Jiao et al., 2020). It’s worth noting that since SNNs use the binary spike for information representation, the multi-valued data preferred by ANNs are encoded into spike train with a time window. When an SNN is mapped to many-core neuromorphic chips, each FCore group needs to perform repeatedly along the T_w_ (length of time window) phases to process a frame image or feature map.

Overall, to end-to-end implement hybrid networks on neuromorphic chips, it requires to not only establish connections among FCore groups that support mixed dataflows, but also coordinate the different requirements of execution phases between ANNs and SNNs.

### Basic Connections for Mixed Dataflow

In order to support mixed dataflows of multi-valued data and spike trains, we design three basic connections based on the fine-grained configuration of input and output modes of the FCores. According to the configuration, four kinds of FCores with different output and input relations can be formed. When the axon and the soma are configured in the same mode (either ANN or SNN mode), the FCore processes pure ANN signals in multi-value data or SNN signals in binary spikes respectively, which can be allocated to perform the calculation in ANN and SNN modules of HNNs. We call such FCores working in the pure-ANN or pure-SNN mode. When data conversion is needed, the axon and the soma are configured to work in different modes, forming hybrid FCores with ANN-input and SNN-output (A2S) or with SNN-input and ANN-output (S2A). These hybrid FCores can be used to implement the conversion between multi-valued data and spike trains, thus supporting hybrid modeling and interaction in HNNs.

**FIGURE 3:**
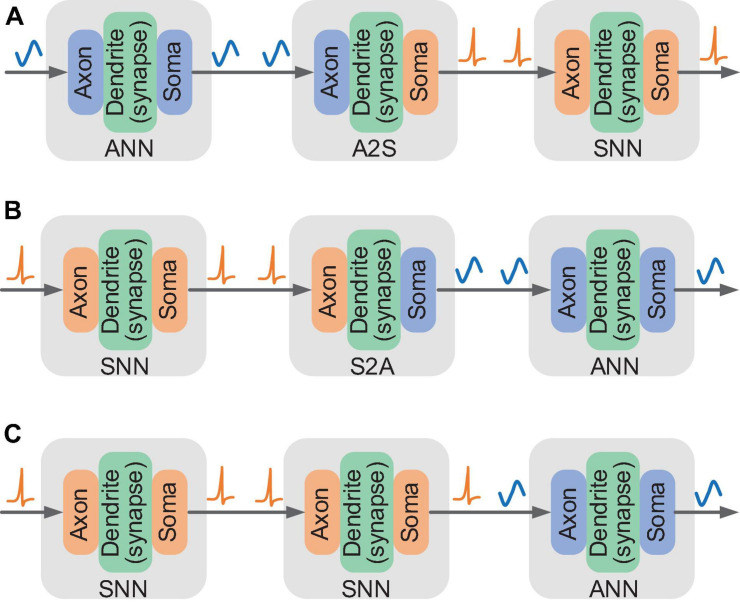
Illustration of three basic connections for mixed dataflow: **(A)** connection supporting the conversion from multi-valued data to spike trains; **(B,C)** connection supporting the conversion from spike trains to multi-valued data.

By virtue of the unified routing infrastructure, these different types of FCores can formulate a variety of basic connections that enable to process single and mixed dataflows. When the soma of the pre-connected FCore and the axon of the post-connected FCore are configured in the same working mode, data can be directly transmitted between them. Figures 3A,B depict the connections that can be used to realize conversion from multi-valued data to spike trains and vice versa, respectively. In Figure 3A, the first and last FCores work in the pure-ANN and pure-SNN modes respectively. The intermediate FCore, working in the A2S mode, receives multi-valued data and converts it into binary spikes via designed internal operations. Similarly, the intermediate FCore working in the S2A mode converts the spike trains to multi-valued data in Figure 3B. In addition, as shown in Figure 3C, an ANN axon can also directly connect with an SNN soma and access neuronal state information from the routing packet. In this connection, the signal conversion occurs during data transmission and reception.

By constructing these basic connections for mixed dataflows, the signal conversion can be naturally performed on the critical data path of FCores without extra devices. In this manner, the signal conversion of the model can be realized on the same carrier as other components. Its effects on the execution performance of the model can be directly displayed.

### Configuration Schemes for Signal Conversion

**FIGURE 4:**
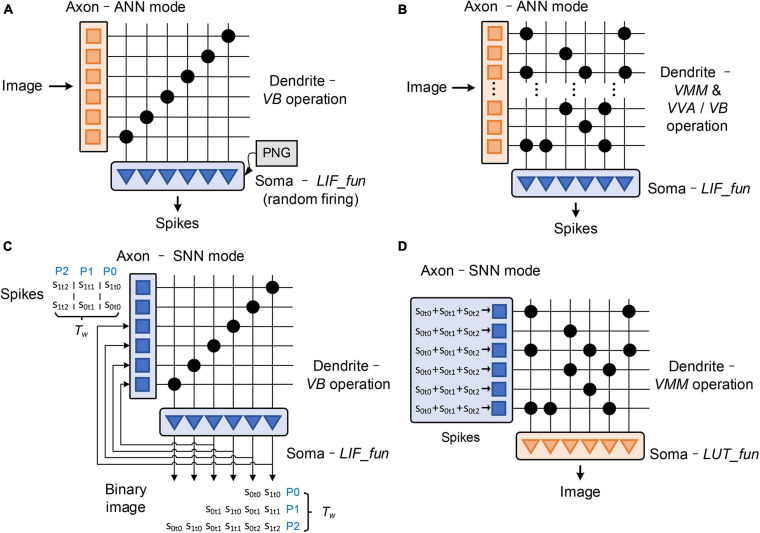
Illustration of the implementation of typical signal conversions: **(A)** probabilistic sampling; **(B)** ANN-SNN encoding layer; **(C)** spatial expansion; **(D)** temporal accumulation.

In general, different signal conversion methods are applied according to the algorithm details. The data interaction between ANNs and SNNs requires signal conversion between spike trains and multi-valued data. The commonly used data conversion methods can be summarized as: probabilistic sampling, ANN-SNN encoding layer, space expansion, and time accumulation (Deng et al., 2020b). Probabilistic sampling and ANN-SNN encoding layer are responsible for the conversion from multi-valued data to spike trains, while spatial expansion and temporal accumulation are responsible for the conversion from spike trains to multi-valued data. We establish configuration schemes for these typical signal conversions (as illustrated in Figure 4), whose operations are mapped into the configurations of the working modes of the building blocks in each FCore.

**Probabilistic sampling** converts multi-valued data to spike trains through an element-by-element operation. At each time phase in the time window, a random vector with the same size as the original multi-valued data is generated. After comparison, the multi-valued data is sampled as binary spikes. The FCores used for realizing probabilistic sampling work in the A2S mode. The axons’ input data is directly transmitted to the soma through *VB* operation. Their somas work in the SNN mode and the random threshold is enabled. Random numbers with uniform distribution are generated as the threshold of membrane potential, so as to realize the sampling of input multi-valued data. The update mode of membrane potential is set to *keep*, so that the multi-valued data can be saved after the first reception, which will be converted into a spike train latter via soma sampling with multiple time phases.

**ANN-SNN encoding layer** can be regarded as a special SNN layer that can process multi-valued input data. Different from probabilistic sampling, the encoding layer adopts a rank-order coding format. Before being converted into spikes, the original multi-valued data is processed by a global calculation in advance, which can be a fully connected calculation (Bellec et al., 2018), a convolution (Wu et al., 2019), or a difference-of-Gaussians (DoG) (Kheradpisheh et al., 2018). The integration results are continuously accumulated onto the membrane potential of the output neuron, and the neuron fires a spike once the membrane potential exceeds the firing threshold at any time phase. In order to reduce redundant integration operations, we implement the encoding layer in two FCore groups: integration FCores and conversion FCores. In integration FCores, the dendrite performs the *VMM* operation at the first time phase in a time window and stores the integration results in the shared memory. The ANN soma of FCores continuously sends out the integration results (or their partial sums) to the conversion FCores, where spikes are generated through the LIF operation in the SNN soma.

**Spatial expansion** directly transfers the spatio-temporal two-dimensional spike pattern into a static binary image. Each “1” in the static binary image corresponds to the existence of a spike in the original spike pattern. In hardware implementation, this spatial expansion method needs to buffer and rearrange data from different time steps. To solve this problem, we establish a self-feedback routing connection in the conversion FCore. The input spikes are shifted and sorted at each time phase. The rearranged spikes are sent to the downstream adjacent ANN axon through multicast routing as binary image data. However, in this conversion method, the scale of the newly generated binary data is proportional to the length of the time window. Consequently, it will consume massive computing and storage resources in the case of a large time window.

**Temporal accumulation** directly accumulates the spike train of each input node within the time window into a multi-valued data. This kind of conversion can be regarded as the reverse process of rate coding, and the converted multi-valued data has the same spatial size as the input spikes. This conversion can be realized by the TFC in axon. Spike accumulation for T_w_ length is enabled in TFC, and the corresponding time factors are configured as 1. In this way, the data transmitted to the dendrite module for integrated calculation is the multi-valued data obtained by accumulation. In the conversion FCore, only the axon is used for signal conversion, and the dendrite and the soma can directly execute the subsequent ANN calculations.

The configuration schemes for these typical signal conversions are summarized in Table 2. In the hybrid networks, the signal conversion operations are mapped into the configuration of the FCore according to the algorithmic computing operations. The model can generate the corresponding special conversion FCore groups in accordance with different requirements to form a connection with hybrid dataflows.

**TABLE 2:** Summary of the configuration schemes for typical signal conversions.

		Probabilistic sampling	Encoding Layer		Spatial expansion	Temporal accumulation
			Integration	Conversion		
Axon	Mode	ANN	ANN	ANN	SNN	SNN (with TFC)
Dendrite	Operation	*VB*	*VMM*	*VVA*/*VB*	*VB*	*VMM*
	Enable time	the first phase	the first phase	always on	always on	always on
Soma	Mode	*LIF_fun* (random firing)	*LUT_fun*	*LIF_fun*	*LIF_fun*	*LUT_fun*
	Enable time	always on	always on	always on	the last phase	the last phase
Membrane potential		*keep*	*keep*	*change*	*change*	*change*

### Global Adjustment of Timing Schedule

As introduced in Section “Execution of Neural Networks on Neuromorphic Chips,” ANNs and SNNs have different pipelining cycles (1 phase in ANN and T_w_ phases in SNN) and need to adjust the timing schedule when combining the ANN layers and SNN layers together. In hybrid models, when an ANN layer runs ahead of an SNN layer, the ANN layer is expected to wait for the SNN layer to execute T_w_ times continuously before transmitting the next data. Similarly, when an ANN layer runs behind an SNN layer, the ANN layer only needs to perform the calculation once after the SNN layer continuously executes T_w_ times. Therefore, it is preferred that in hybrid models, the ANN only starts at a suitable time for effective calculation and data transmission, instead of performing the same operation phase by phase repeatedly. Hence, we use the configurable phase pattern introduced in Section “Adjustable Timing Schedule” to realize a global timing adjustment of the timing schedule of the FCores.

**FIGURE 5:**
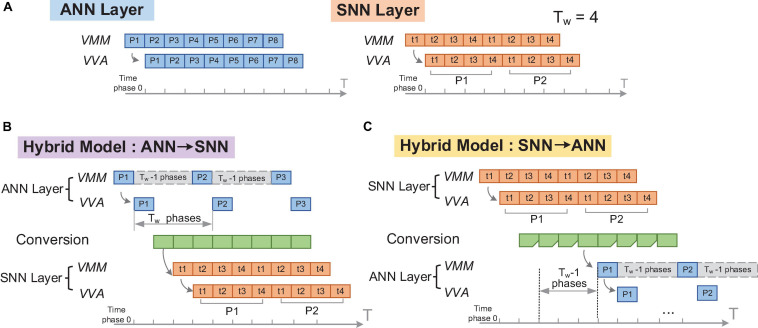
Illustration of the global adjustment of timing schedule: **(A)** original ANN and SNN layers; **(B)** timing configuration of an SNN layer running after an ANN layer; **(C)** timing configuration of an ANN layer running after an SNN layer.

We demonstrate this global adjustment mechanism in Figure 5. Figures 5B,C illustration the situation of “ANN layer to SNN layer” and “SNN layer to ANN layer” respectively. In both situations, the time step is set to include T_w_ time phases. In each time step, the FCore groups corresponding to the SNN calculation execute continuously, while the FCore groups corresponding to the ANN calculation only start at the first phase. Therefore, the phase patterns of these two types of FCore groups will be configured as #on_ phases = Tw, #off_ Phases = 0 and #on_ phases = 1, #off_ phases = Tw-1 respectively. Due to the delay of data transmission, different FCore groups will have different start-up time. Generally, this start-up delay is the number of FCore groups that need to pass before data arrives. However, in the mapped structure, as long as there exists an FCore group for ANN-SNN conversion, the subsequent start-up delay needs to increase the extra time required by the ANN to wait for SNN to process data (i.e., T_w_-1 time phases, see Figure 5C). After setting the correct start-up delay, the ANN and SNN in the hybrid model can continually process each frame of input data in a pipelined manner, “step” by “step.”

As shown in the Table 2, following the same method, the dendrite and the soma in the conversion FCores are also configured to have different phase patterns to deal with the intra-FCore mixed dataflow. These timing patterns are eventually transformed into the configuration of timing registers in each FCore. Such a global timing adjustment can not only keep the original pipeline mechanism, but also reduce redundant calculations.

## Experimental Results

### Experimental Setup

We have built some examples of hybrid networks to verify our mapping framework and illustrate the implementation results. We chose MNIST (LeCun et al., 1998) and NMNIST (Orchard et al., 2015) data sets to demonstrate the proposed conversion approach. Each digit sample in MNIST is a 28 × 28 grayscale image and NMNIST is a neuromorphic version of MNIST with a spike pattern size of 34 × 34 × 2 at each time step. As for the network structure, we chose the fully connected structure of input-512-512-10 and the convolutional neural network structure of LeNet (LeCun et al., 1998) (input-6c5-AP2-16c5-AP2-120-84-10). The overall settings of input data, network structure and signal conversion method are shown in the Table 3.

**TABLE 3:** Network models used in the experiments.

Dataset	Structure	Conversion Method	Name
MNIST (28 × 28)	MLP	Probabilistic Sampling	Model 1
		Encoding Layer	Model 2
	LeNet	Probabilistic Sampling	Model 3
		Encoding Layer	Model 4
NMNIST (34 × 34 × 2)	MLP	Spatial extension	Model 5
		Temporal accumulation	Model 6
	LeNet	Spatial extension	Model 7
		Temporal accumulation	Model 8

**FIGURE 6:**
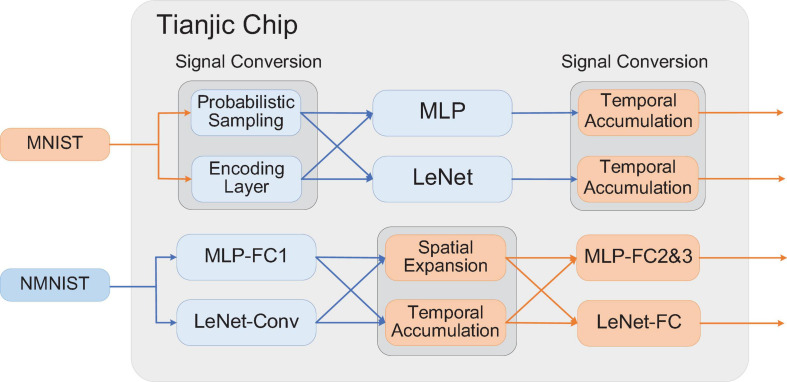
Illustration of the configuration of the models.

Figure 6 shows the settings of signal conversion in these models. From Model 1 to Model 4, the signal conversion from multi-value data to spike trains happens in the first layer of the network. At the output of these models, the spikes within the time window are accumulated and converted into multi-valued data to obtain the classification results. In Model 5 and Model 6, the signal conversion from spike trains to multi-valued data occurs in the connection between the first and second hidden layers. In Model 7 and Model 8, the signal conversion is performed between the last pooling layer and the fully connected classifier.

We developed a mapping compiler in python to automatically implement the partition of networks, generation of conversion FCores, and global timing adjustment in our mapping framework. We used a direct quantization method for these hybrid models. We first loaded a pre-trained model with FP32 weights, and then followed the quantization method in Yang et al. (2020) to re-train the model by quantizing the weights into INT8 precision during each weight update. After this process, we can obtain the resource utilization report of network and the binary file for the chip configuration. At last, the configuration file is downloaded into the chip for execution, where the latency and power consumption of network execution can be measured accurately. We use a single-chip PCB equipped with an Altera Cyclone 4 FPGA as the test board. The input data is pre-stored in an SDRAM on the board, and injected into the chip through FPGA whiling testing. At 300 MHz clock, 16.8 μs is needed for a time phase.

### Analysis of Resource Consumption of Various Hybrid Models on Tianjic

**FIGURE 7:**
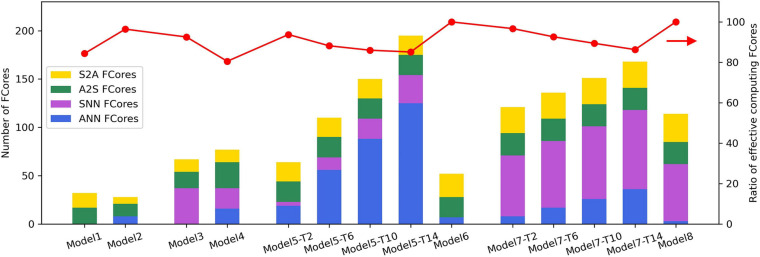
Resource consumption of the example hybrid models.

After mapping, these hybrid network models are transformed into the connections between FCores with four different input and output types, as described in Section “Basic Connections for Mixed Dataflow.” Figure 7 shows the resource utilization of different types of FCores in each hybrid model. When mapping the SNN part of these hybrid models, according to the method in Pei et al. (2019); Deng et al. (2020a), we transfer the partial sums calculated by *VMM* in the form of multi-valued data. This method can avoid the decrease of accuracy caused by the fan-in limitation of FCores in the mapping process. Therefore, when the number of input neurons in an SNN layer exceeds the fan-in of FCore, there exist S2A-type *VMM* FCores and A2S-type *VVA* FCores in the mapping result. Additionally, we define the ratio of effective computing FCores as the ratio of the number of FCores that perform network computing and the number of the total FCores. The larger the ratio of effective computing FCores is, the smaller the extra cost of signal conversion in hybrid models consumes.

In a connection with the signal conversion from multi-valued data to spike trains, the resource consumption required by the probabilistic sampling and ANN-SNN encoding layer is determined by the number of input and output neurons in the connection, respectively. In the MLP structure, the conversion takes place in a 784-512 connection. Therefore, compared with Model 2, Model 1 uses more A2S-type FCores. Similarly, in the LeNet structure, the size of input and output in the connection with signal conversion is 28 × 28 × 1 and 24 × 24 × 6, respectively, which leads to Model 4 consuming more conversion FCores than Model 3. In the implementation of probabilistic sampling and ANN-SNN encoding layer, the signal conversions are carried out through additional FCores. From Figure 7, we can see that the ratio of effective computing FCores in Model 1∼Model 4 are 84%, 96%, 92%, and 81%, respectively. This result also indicates that with the increase of the scale of converted signals, the proportion of the FCores that undertake the network computation in the hybrid model decreases.

In the implementation of spatial expansion, the conversion FCores work in the SNN mode to buffer and rearrange input spikes. As mentioned above, the FCores consumed by spatial expansion will increase with the increase of the SNN time window. To observe this trend, we show the resource consumption of Model 5 and Model 7 when T_w_ equals 2, 6, 10, and 14 respectively (shown as -T2, -T6, -T10 and T14 in Figure 7). As we can see, with the increase of T_w_, the number of SNN-type FCores increases concomitantly, which is caused by the increase of the number of occupied conversion FCores. Furthermore, due to the growth of the scale of the converted binary image, the number of ANN-type FCores that are used to perform subsequent ANN calculations also increases. It is observed that, under the joint influence of these two kinds of growth, the ratio of effective computing FCores of the network finally shows an obvious downward trend. In the implementation of spatial expansion, only axons in the FCores are used, which does not affect the subsequent calculation of dendrites and somas. As a result, the ratios of effective computing FCores are both 100% in Model 6 and Model 8. Regardless of the types of FCores, the resource consumption of these two models is the same as that of a single-paradigm network with the same structure.

### Comparison of Performance on Tianjic and GPU

We tested the implementation of these hybrid models on GPU (Nvidia RTX 2080Ti) and the Tianjic chip, respectively, and summarized the outcomes in Table 4. The time window of SNNs in these hybrid models was set as 10. These hybrid models ran on GPU with the default FP32 precision. While running on Tianjic chip, all the weights and output activations were quantized to INT8. It is observed that there is no evident difference in recognition accuracy between the fixed-point network implemented on the Tianjic chip and the floating-point network running on GPU. In some models (i.e., Model 2 and Model 7), the accuracy on the Tianjic chip was slightly improved, which is owing to the regularization effect of quantization. Generally speaking, the accuracy of the model varies within 0.15%, which is almost negligible.

**TABLE 4:** Execution performance of different implementations on Tianjic and GPU.

	GPU (Nvidia RTX 2080Ti)		Our Implementation	
	Acc. (%)	Latency (ms)	Acc. (%)	Latency (ms)
Model1	98.70	6.84	98.69	0.286
Model2	98.20	6.02	98.22	0.269
Model3	99.15	14.27	99.15	0.319
Model4	99.19	13.22	99.13	0.319
Model5	98.43	3.56	98.41	0.252
Model6	98.36	3.22	98.30	0.269
Model7	98.27	8.79	98.28	0.302
Model8	98.97	8.87	98.85	0.319

It is worth noting that, while running on Tianjic, all these models consume about 300 ms latency to process one frame of data. Compared with GPU, the processing speed is increased by an average of 20 times. This is mainly due to the high computational parallelism of the many-core architecture in the Tianjic chip. In all these models, the average power consumption is below 400 mW, verifying the advantage of low power consumption compared with GPU (usually with a dynamic power of 1∼100 W).

Through the comprehensive comparison, we can conclude that when implemented on the Tianjic chip, the hybrid models not only obtain nearly lossless accuracies, but also exhibit significant advantages of low processing latency and low power consumption. In the next section, we will further analyze the energy consumption of different parts that are responsible for ANN calculations, SNN calculations, and signal conversions, respectively, in the hybrid models.

### Analysis of Energy Consumption

**FIGURE 8:**
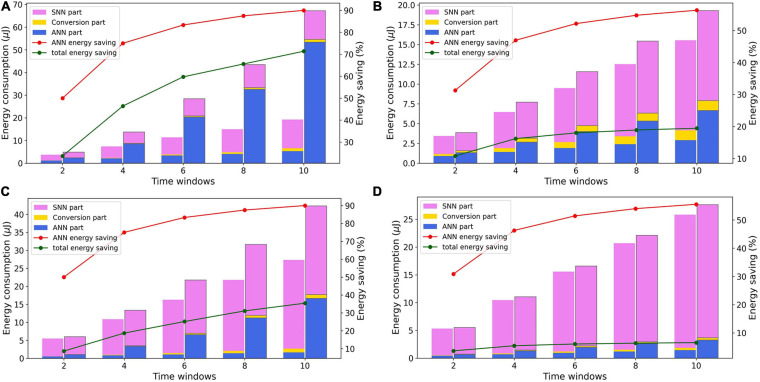
Variation of dynamic energy consumption distribution with time window of panels **(A)** Model 5, **(B)** Model 6, **(C)** Model 7, and **(D)** Model 8.

Taking Model 5 to Model 8 as examples, we analyze the distribution of dynamic energy consumption and its change along with the time window. The dynamic energy distribution in these hybrid models when T_w_ equals 2, 4, 6, 8, and 10 are visualized in Figure 8. For each T_w_ value, the dynamic energy consumption transformation before and after the global adjustment of timing schedule are also plotted.

The Global adjustment of timing schedule can reduce the total dynamic energy consumption of network by reducing redundant ANN operations. Evidently, as the time window increases, the energy saving of the ANN calculations under the same conversion method retains the same. Model 5 and Model 7 use the spatial extension method and the input size of the ANN layer will increase along with T_w_. As shown in Figure 7, the increase of the input size will consume more ANN FCores and thus more dynamic energy consumption. Nevertheless, due to the reduction of redundant operations brought by the global timing adjustment, the dynamic energy saving of ANN FCores in both Model 5 and Model 7 increases from about 50% to about 90% as T_w_ increases. In Model 6 and Model 8, after the global timing adjustment, there are still some VMM operations that need to be executed repeatedly in the conversion FCores to drive the shift of the axon’s spike buffer. Along with the increase of T_w_, these repeated executions lead to the increase of ANN FCores’ dynamic energy consumption in the adjusted models. As a result, Model 6 and Model 8 have a lower dynamic energy saving than Model 5 and Model 7, which increases from about 31% to about 56%. Additionally, due to the different proportion of ANN and SNN, the total energy saving also differs in different models. In a hybrid network, the larger the proportion of ANN calculations is, the more energy can be saved after global timing adjustment.

After the global timing adjustment, when T_w_ equals 2, the energy consumptions required for signal conversion in Model 5 to Model 8 accounts for a small part, which are 7.3%, 1.6%, 1.3%, and 0.9%, respectively. With the increase of T_w_, these ratios all increase slightly. In the method of spatial expansion, because the number of conversion FCores is also increasing, the proportion will rise a little faster. But these ratios do not exceed 10% in the end. Furthermore, the difference in the dynamic energy consumed by SNN and ANN calculations is not as large as the difference in the number of FCores (about 6: 1 and 10: 1 in MLP and LeNet, respectively), indicating that the advantage of the computational sparsity in SNNs is well utilized in hardware execution.

## Conclusion and Discussion

In this paper, we propose a systematic solution of implementing various hybrid networks on many-core neuromorphic chips through software-hardware cooperation. Based on the abstraction of the Tianjic chip, we summarize that the fine-grained configurable basic units, unified communication format, and adjustable timing schedule provide the hardware foundation for implementation of hybrid models. On this basis, we propose an end-to-end mapping framework to facilitate implementation of hybrid models on hardware. By constructing basic connections for mixed dataflows, signal conversions are performed on the critical data paths of FCores without requiring additional devices. The configuration schemes for the typical four types of signal conversions are designed and proved to promote the mapping of the operations in hybrid models into FCores in the same way as that of the networks’ computing operation. The global adjustment of timing schedule not only ensures the continuity and correctness of data transmission and processing in the network, but also reduces the energy consumption caused by the repeated redundant calculations. Furthermore, we built a tool chain to automatically implement the mapping framework. By mapping typical hybrid models to the Tianjic chip, we demonstrate that these models not only obtain almost lossless accuracy compared with the general computing platform, but also exhibit significant advantages of low execution latency and low power consumption. The results of the experiment show that although the implementation of signal conversion in HNNs generates additional resource overhead, the overall energy consumption of the network can be significantly reduced by disabling repeated redundant operations.

Generally, SNNs have rich coding schemes to encode information in spatio-temporal domain, including rate coding schemes (Deng et al., 2020b) and temporal coding schemes like rank-order coding schemes (Tang et al., 2020), inter-spike interval based (Dong et al., 2019) and time-to-first spike based encoding schemes (Liu and Yue, 2017; Mostafa, 2017). Our framework can effectively support the rate coding and rank-order coding scheme for SNNs and in principle can support the temporal coding schemes via the unified communication format. The unified communication format can directly transmit the temporal information through its data segment (i.e., 8-bit *Fire_data* in the routing packet) to support the inter-spike interval based and time-to-first spike based encoding schemes. To effectively support these temporal coding schemes, efficient capture of the absolute or interval time information of each neuron’s output spikes is necessary and requires further improved hardware design.

With the increasing complexity of tasks and the deepening of artificial intelligence research, it is expected that more and further cross-paradigm fusions of ANNs and SNNs will emerge. Hence, we are convinced that our proposed end-to-end hardware implementation method will provide a systematic solution to map hybrid models onto neuromorphic chips, and provide guidance for further development of hybrid neural models. Moreover, through the modeling abstraction of hardware characteristics, mapping mechanisms can be established to fully explore the potential of the hardware carrier and support more complex algorithm models. Through the iteration of hardware-software co-optimization, it is highly possible to develop a general brain-inspired computing platform that can handle more complex tasks.

## Data Availability Statement

The original contributions presented in the study are included in the article/supplementary material, further inquiries can be directed to the corresponding author/s.

## Author Contributions

GW proposed the idea, designed and did the experiments, and wrote the manuscript. GW and YW conducted the algorithm modeling work. GW, SM, and JP conducted the design and implementation of the hardware testing platform. GW and SM contributed to the analysis and interpretation of results. RZ led the discussion and revised it. LS directed the project and provided overall guidance. All authors contributed to the article and approved the submitted version.

## Conflict of Interest

The authors declare that the research was conducted in the absence of any commercial or financial relationships that could be construed as a potential conflict of interest.
